# Trends of admission and predictors of neonatal mortality: A hospital based retrospective cohort study in Somali region of Ethiopia

**DOI:** 10.1371/journal.pone.0203314

**Published:** 2018-09-14

**Authors:** Abdifatah Elmi Farah, Abdulahi Haji Abbas, Ahmed Tahir Ahmed

**Affiliations:** 1 Independent Consultants, Jigjiga, Ethiopia; 2 Public Health Department, College of Medicine and Health Sciences, Jigjiga University, Jigjiga, Ethiopia; National Institute of Health, ITALY

## Abstract

**Background:**

In Ethiopia, the trend of neonatal mortality showed slow pace of reduction from 2000–2016 compared to the reduction in infant and under-five mortality over the same period. This study aimed at unpacking the trends of admission, specific causes and rate of neonatal mortality as well as predictors of neonatal mortality at a general hospital in Somali region of Ethiopia.

**Methods:**

A hospital based retrospective cohort study was conducted from 25^th^ of May 2017to 10^th^ of June, 2017. All the new-borns admitted in the neonatal Intensive Care Unit of the hospital were reviewed over three years period (Aug2014-May2017). Data were cleaned and exported to SPSS version 20 and both descriptive and analytical analysis were executed. The level of significance was set at P<0.05. Binary logistic regression was used to produce summary of statistics including crude and adjusted odds ratio and 95% confidence intervals.

**Results:**

This study reviewed a total of 792 new-borns below the age of 28 days admitted in the NICU of Karamara hospital over a period of three years (August, 2014 to May, 2017). The mean birth weight was 2733 ± 740 g and neonates with normal birth weight stood at 64%. Seven hundred forty seven new-borns (94.3%) were discharged alive while 45 (5.7%) new-borns died in the course of hospitalization making a Neonatal Mortality Rate (NMR) of 5.7% (57 per 1000 live births), and 96 percent of these deaths were early neonatal deaths that occurred in the first one week of life (i.e. <7 days).After all variables which had an association with neonatal mortality (P <0.05) were entered in to a multivariate logistic model to control the effect of confounders: prematurity (AOR = 0.492(0.253, 0.957), P = 0.037) and average length of stay below two days (AOR = 0.418(0.186, 0.936), P = 0.034) were independently associated with neonatal mortality showing protective effect.

**Conclusion:**

The causes of neonatal death reported in this study are preventable, the neonatal mortality rate is high compared to the national and regional figures. Prematurity and shorter length of stay in the NICU of less than two days showed independent association with neonatal mortality. This calls for more work along the continuum of care, improving the quality of care, early transfer of sick neonates to the NICU along with scaling up establishment of the NICUs in other hospitals of the region.

## Introduction

Substantial progress has been made in reducing child deaths globally including sub-Saharan Africa—the region with the highest under-five mortality rate in the world. Similarly, the global neonatal mortality rate fell from 36 deaths per 1,000 live births in 1990 to 19 in 2015. In absolute terms, the number of neonatal deaths declined from 5.1 million to 2.7 million over the same period. However, between 1990 and 2015, neonatal mortality showed a slower the decline than that of post-neonatal under-five mortality i.e. 47 percent compared to 58 percent globally [1, 2].

Ethiopia has a similar story. It has made considerable progress in reducing under-five mortality rate. According to the 2014 UN Inter- agency Group for Child Mortality Estimation (UN-IGME) report, Ethiopia is one of the eight high-mortality countries where the under-five mortality declined by two thirds, thus achieving MDG4 three years ahead of the 2015 deadline [3].

Nonetheless, the mortality reduction is not uniform across the different childhood age groups as well as geographic and socio-demographic population groups [4]. There has been a steady decline in infant, child, and under-five mortality over the last 16 years (2000–2016). In particular, under-five mortality rates declined from 166 to 67 deaths per 1,000 live births in 2016. Similarly, infant mortality decreased from 97 to 48 deaths per 1,000 live births in the same period whilst the neonatal mortality decreased from 54 to 29 per 1000 live births [5]. This shows that the trend of neonatal mortality reduction in Ethiopia is moving at a slow pace compared to the reduction of infant and under-five mortality over the same period[3–7].

Currently, Ethiopia is one of the top five countries contributing more than half of the neonatal deaths globally [8]. Children are still dying of diseases which could have been prevented and/or treated easily with low cost and effective interventions [1]. Over two-third of childhood deaths in Ethiopia are mainly due to infections, neonatal conditions and malnutrition. The neonatal causes of death are prematurity, low-birth-weight, infections, asphyxia (lack of oxygen at birth) and birth trauma which together account for nearly 80% of deaths in this age group [6, 9].

About 44 percent of these childhood deaths occur within the first 28 days of life, thus increasingly accounting for a larger proportion of the under five deaths[2, 6, 10]. Moreover, 79 percent of neonatal deaths occur during early neonatal period (0–6days) of which 41 percent is in the first 24 hours of birth where as 21 percent happen in late neonatal period (7-27days). Mortality differences exist by place of residence, region, education of the mother, and household wealth with the Somali region having a perinatal mortality rate of 42 per 1000 births [7]. Additionally, disparities in coverage of high impact child survival interventions also remain high across Ethiopia’s administrative regions, and between residents of urban and rural areas. In two emerging administrative regions of Ethiopia (Afar and Somali), a significant majority of women and children receive 2 or less out of 8 essential RMNCH interventions [11].

A facility based study in Ethiopia which reviewed 3,789 new-borns below the age of seven days reported that an early neonatal mortality rate of 23.3 percent and 96.6 percent of these deaths occurred during the first three days of life. First born, preterm and low-birth weight infants accounted for 20.5%, 52.5% and 59.3% of neonatal deaths respectively[12].

Generally, real progress in reducing deaths of new-born babies in a country with higher neonatal mortality like Ethiopia demands a higher coverage of optimally standard neonatal services with special focus on the poorest segment of the population and at the time of greatest risk, which is at birth and in the first few days of life [13]. Neonatal mortality is becoming increasingly important not only because of its share of under-five deaths has been increasing, but also the health interventions needed to address the major causes of neonatal deaths generally differ from those needed to address other under-five deaths and are closely linked to those that are necessary to protect maternal health[13].

It is very recently that new-borns received policy and programmatic attention from the health system. Following advocacy efforts, new-born health is now one of Ethiopia’s top priorities in the 2015–2020 Health Sector Transformation Plan and a new revised child survival and new-born strategy is issued[14] which puts New-born Intensive Care Unit (NICU) among the list of high impact child survival interventions being implemented to reduce neonatal deaths immediately after child birth–the most vulnerable period for neonates [4].

It is a national norm to ensure that there is a functional newborn corner in each health center, a well-spaced NICU with four to six nurses in each hospital. However, the coverage of these interventions remained generally very low in Ethiopia and particularly in Somali region [4, 13] whereby only one hospital is providing full-fledged NICU service despite an estimated 10–15% of newborn babies requiring the service [15]. Some of the common challenges for newborn health in Ethiopia and Somali region in particular include: poor health system governance, weak and uncoordinated referral system, widely prevailing and deeply-rooted cultural practices, and low skill among health extension workers in providing essential neonatal care services. Moreover, Other supply side challenges include ill-equipped health care facilities, shortage/lack of skilled health workers, sophisticated equipment, and expensive drugs needed to provide emergency obstetric and newborn care[11].

Hence, this study aims at unpacking the trends of admission, specific causes and rate of neonatal mortality as well as predictors of neonatal mortality at a general hospital in Somali region of Ethiopia. The findings of this study will be used for evidence based newborn health programming.

## Materials and methods

### Study area

This study was done at a Neonatal Intensive Care Unit in Karamara General Hospital located in Jigjiga -Somali region of Ethiopia. The NICU has three rooms with 12 beds in total: one room for Neonatal Intensive Care Unit, one for kangaroo mother care and another for septic neonates. It is the only hospital providing fully functioning NICU service in the region.

### Study duration

The study was conducted from 25^th^ of May to 10^th^ of June, 2017.

### Study design

Hospital based retrospective cohort was used to review 792 new-borns admitted in the NICU of Karamara hospital from August 2014 to May, 2017.

### Study subjects

New-borns below the age of 28 days who were admitted in the NICU from August 2014 to May 2017 were considered in this study.

### Exclusion criteria

Individual recordings in the register which were improperly filled were excluded.

### Data collection technique

The source of data for this study was the NICU register at karamara hospital which consisted of new-born information recorded at admission such as date of admission, age, weight of the child, status at birth, diagnosis, treatments given, outcome status and records of maternal information like parity, ANC follow up, gestational age and mode of delivery. All these data were collected using a uniform extraction format developed by taking in to account all the relevant variables in the standard NICU registration book.

### Method of data analysis

The raw data were entered in to excel and checked for incomplete and inconsistent data, then missing values were excluded before exporting to SPSS version 20. Cross tabulation of admission and death events and graphs were used to summarize and present the data. Neonatal mortality rate was calculated using the total neonatal deaths recorded at the NICU divided by the total number of new-borns admitted at the NICU in the three years reviewed. The causes of death were analysed by socio-demographic, maternal and new-born characteristics. The level of significance was set at P<0.05. Binary logistic regression was used to produce a summary statistics of proportions including crude and adjusted odds ratio and 95% confidence intervals. A bivariate analysis using the Chi-square test or Fisher exact test, where appropriate, was performed to determine predictors of neonatal hospital mortality pertaining to neonatal characteristics for each variable one at time. Statistically significant (P<0.05) variables were then entered in to multiple logistic regression to eliminate possible confounders.

### Data quality assurance

For data collection purposed, we recruited a nurse and trained him with daily supervision. Feedback and corrective actions were taken upon review of the daily submitted data. Cross-checking with the source registration book was applied for any observed incompleteness, error, and/ ambiguities in recording.

### Study variables:

**Dependent variable**:

- Neonatal outcome (Survived, Died)

**Independent variables**:

- **Socio-demography**
  ○ age of the mother/neonate;
- **Maternal/Neonatal factors**
  ○ ANC, parity, maternal HIV status, gestational age, multiple pregnancy, mode of delivery, status at birth, birth weight, respiratory distress, prematurity, sepsis, perinatal Asphyxia, anaemia, congenital malformation, Meconium aspiration syndrome, Jaundice, feeding.

### Ethical clearance

This study was exempted from ethical clearance by Ethics Review Committee of College of Medicine and Health Sciences of Jigjiga University on the 24^th^ of March 2017 based on a provision under the Ethiopian Research Ethics Review Guideline (5^th^ edition September, 2014). Additionally, anonymity was kept to preserve personal identifiers and maintain confidentiality.

## Results

This study reviewed a total of 792 new-borns below the age of 28 days who were admitted in the NICU at Karamara Hospital over a span of three years (August, 2014 to May, 2017). While all neonates were born in the hospital, 95 percent of them were below six days at the time of admission of which about three-fourth were first born. The mean birth weight was 2733 ± 740 g (range: Between 800 and 6000 g) and neonates with normal birth weight stood at 64%.

Seven hundred forty seven new-borns (94.3%) were discharged alive while 45 (5.7%) died in the course of hospitalization making a Neonatal Mortality Rate (NMR) of 5.7% (57 per 1000 live births). Ninety-six percent of these deaths were early neonatal that occurred in the first one week of life (<7 days) (Table 1).

**Table 1:** Frequency distribution of new-born factors in the NICU of Karamara Hospital, Somali region of Ethiopia from 2014 to 2017.

Newborn variables		Discharge		Total	P-value
		Died (%), n = 45	Survived (%), n = 747		
Birth weight (grams)	< = 1500	10(18.5%)	44(81.5%)	54	.998
	1501–2499	11(5.7%)	183(94.3%)	194	.998
	2500–3999	23(4.7%)	467(95.3%)	490	.998
	4000+	0	24(100%)	24	1
Age during admission	< = 6	43(5.7%)	707(94.3%)	750	.792
	7+	2(4.8%)	40(95.2%)	42	1
Prematurity	No	31(4.9%)	604(95.1%)	635	
	Yes	14(8.9%)	143(91.1%)	157	.054
Suspected Sepsis	No	34(5.5%)	581(94.5%)	615	1
	Yes	11(6.2%)	166(93.8%)	177	.728
Respiratory Distress	No	36(5.7%)	593(94.3%)	629	1
	Yes	9(5.5%)	154(94.5%)	163	.921
Perinatal Asphyxia	No	44(5.8%)	721(94.2%)	765	1
	Yes	1(3.7%)	26(96.3%)	27	.654
Congenital malformation	No	45(5.8%)	731(94.2%)	776	.999
	Yes	0	16(100%)	16	
Meconium Aspiration	No	33(6.2%)	503(93.8%)	536	1
	Yes	12(4.7%)	244(95.3%)	256	.405
Clinical Jaundice	No	44(5.6%)	737(94.4%)	781	1
	Yes	1(9.1%)	10(90.9%)	11	.627
Feeding	Human M	42(5.4%)	736(94.6%)	778	.326
	Mixed	2(28.6%)	5 (71.4%)	7	.522
	Other M	1(14.3%)	6(85.7%)	7	1
ALS	< = 2	22(9.5)	209(90.5%)	231	.049
	3–7	14(3.9%)	347(96.1%)	361	.722
	8+	9(4.5%)	191(95.5%)	200	1

**Fig 1:**
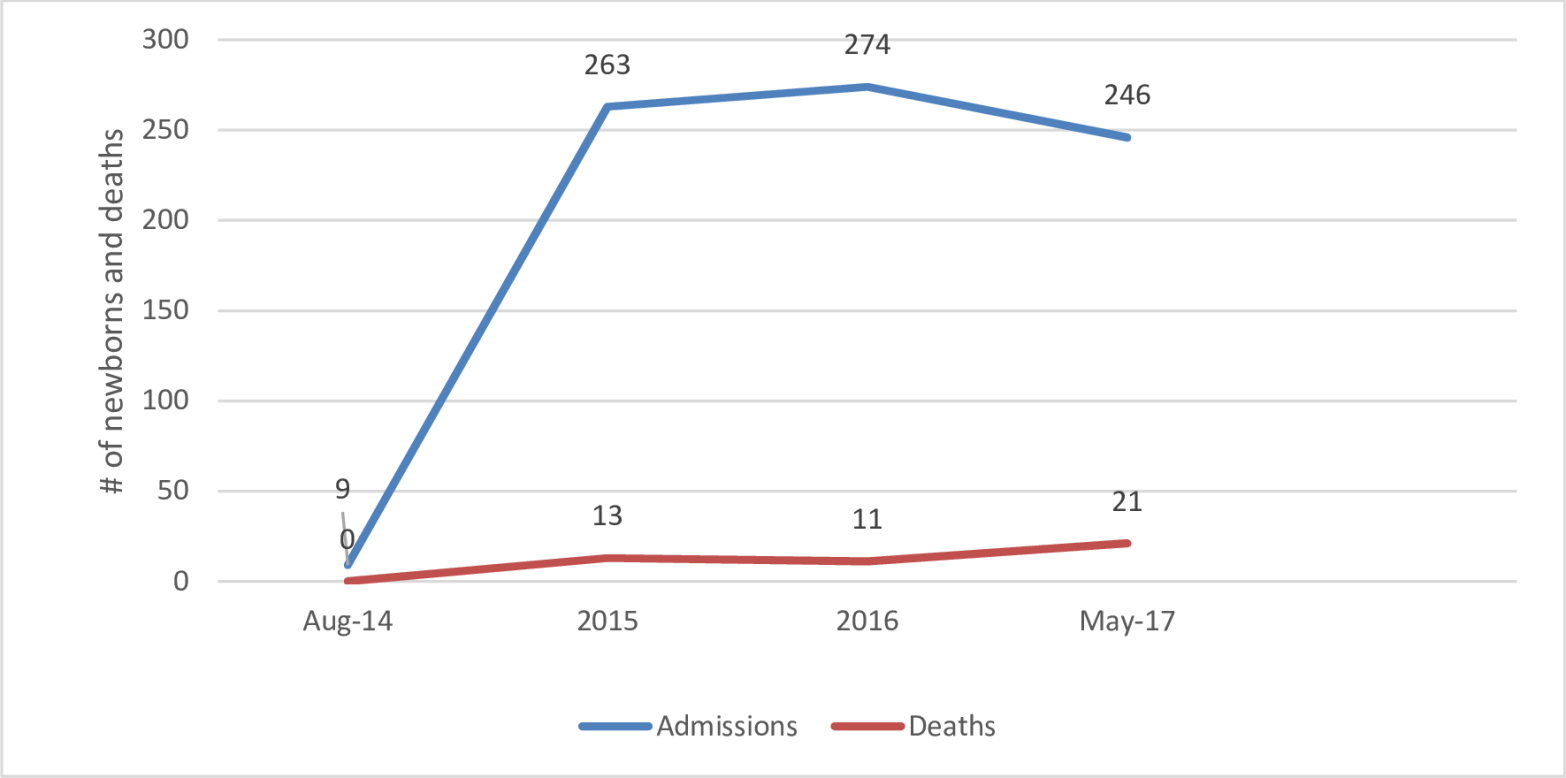
Trends of neonatal admissions and deaths in NICU of Karamara hospital from August 2014 to May 2017.

The trend of admission has risen since the neonatal intensive care unit was established in December, 2014. This marked increment ranged from nine cases in late 2014 to 246 in the first five months of 2017. Early transfer of high risk and premature neonates from the hospital maternity contributed a lot in the admission (Fig 1). The leading causes of admission were: prematurity, sepsis, respiratory distress and meconium aspiration syndrome. Similarly, the causes of death were prematurity, suspected sepsis, meconium aspiration syndrome and respiratory distress/perinatal asphyxia which accounted for 31.1%, 24.4%, 24.4% and 20% respectively.

With regard to maternal factors shown in (Table 2), the majority (71%) were Primi-paras who had ANC (89%) during their pregnancy and 91% of them delivered spontaneously while their pregnancy was at term.

**Table 2:** Frequency distribution of maternal factors in the NICU of Karamara hospital, Somali region of Ethiopia from 2014 to2017.

Maternal variables		Discharge		Total	P-value
		Died (%) n = 45	Survived (%), n = 747		
Mode of delivery	Assisted vaginal	0	12 (100%)	12	1
	C/S	4 (6.9%)	54 (93.1%)	58	.999
	SVD	41(5.7%)	681(94.3%)	722	.999
ANC visit	No	8 (9%)	81 (91%)	89	
	Yes	37 (5.3%)	666 (94.7%)	703	.158
Maternal HIV status	- ve	45(5.7%)	744 (94.3%)	789	.999
	unknown	0	3 (100%)	3	1
Multiple birth	No	45(5.7%)	740 (94.3%)	785	1
	Yes	0	7 (100%)	7	.999
Gestational age	< = 32	18(5.7%)	296 (94.3%)	314	.999
	33–37	12(10.2%)	106 (89.8%)	118	.999
	37–42	15(4.2%)	342 (95.8%)	357	.999
	>42	0	3 (100%)	3	.999
Parity	1	31(5.5%)	533 (94.5%)	564	.741
	2	5(6%)	78 (94%)	83	.956
	3+	9(6.2%)	136 (93.8%)	145	1

The association between maternal and new-born variables with neonatal mortality using binary logistic regression one at a time indicates that neonates with an average length of stay of less than two days had less odds of death (COR = 0.448(0.201, 0.996), P = 0.049) as compared to those with longer length of stay. Prematurity (P = 0.054) was at the border-line of significance thus considered to go for the next step of adjustment not to miss any association (Table 3).

**Table 3:** Bivariate logistic regression of maternal and new-born factors in the NICU of karamara hospital in Somali region of Ethiopia from 2014 to 2017.

Variables		Discharge		P-value	COR	95% C.I. for COR	
		Died (%) n = 45	Survived (%), n = 747			Lower	Upper
Prematurity	No	31 (4.9%)	604 (95.1%)	1			
	Yes	14 (8.9%)	143 (91.1%)	.054	.524	.272	1.011
ANC visit	No	8 (9%)	81 (91%)				
	Yes	37 (5.3%)	666 (94.7%)	.158	1.778	.800	3.950
Average Length of Stay (ALS)	< = 2	22 (9.5)	209 (90.5%)	.049	.448	.201	.996
	3–7	14 (3.9%)	347 (96.1%)	.722	1.168	.496	2.748
	8+	9 (4.5%)	191 (95.5%)	1			

As can be seen in (Table 4), after variables which had an association with neonatal nortality (P <0.05) were entered in to a multivariate logistic model to control the effect of confounders, prematurity (AOR = 0.492(0.253, 0.957), P = 0.037) and average length of stay below two days (AOR = 0.418(0.186, 0.936), P = 0.034) were independently associated with neonatal mortality and showed a protective effect on neonatal death in the NICU.

**Table 4:** Multivariable logistic regression of maternal and new-born factors in the NICU of Karamara hospital in Somali region of Ethiopia from 2014 -to2017.

Variables		P-Value	AOR	95% C.I. for AOR	
				Lower	Upper
Prematurity	Yes	.037	.492	.253	.957
	No	.000	26.443		
Average length of stay	< = 2	.034	.418	.186	.936
	317 days	.800	1.117	.473	2.638
	> = 8+	.010			

## Discussion

This study found a neonatal mortality rate of 5.7% (57 per 1000 live births) in NICU of the study hospital. This is comparable to other facility based studies done in Portugal and Burkina- Faso which reported a rate of 5.7% [16] and 5.3% [17] respectively. In contrast, the 2016 EMONC assessment reported the presence of a lower rate in Somali region of Ethiopia [18] while studies in South Africa reported 3.1% and 3.8% in 2007 and 2008 respectively[19]. Additionally, reports of higher mortality rate were found from Qatar (6.5% between 2002 and 2006 [20], England (8.1% between 2008 and 2010) [21], Nigeria (14.2% between June 2012 and May 2013) [22], Uganda (26–29% in 2012) [23], Rwanda (50% between 2012 and 2014) [24] and Cameroon (15.7%) [25]. The above variations in mortality rates could be explained by the differences in sample size, study settings, timing of the study and causes of death which vary between developed and developing nations. While new-borns typically die from unpreventable causes such as congenital abnormalities in developed countries, majority of infants in developing countries die from preventable conditions, including infections, birth asphyxia, and prematurity[10].

This study also found a higher percentage (96%) of early neonatal death which is in turn related to the very high percentage of new-borns that were below six days of life at the time of admission in the NICU. This is in agreement with studies that show the burden of neonatal deaths occur in the first one week particularly the first 24 hours of life [7, 12, 26, 27] which highlight conditions related to labour, intra-partum and immediate new-born care practices.

The leading causes of admissions and subsequent deaths in the NICU were found to be prematurity, sepsis, meconium aspiration syndrome and respiratory distress/perinatal asphyxia. Although this is consistent with studies done in Ethiopia and abroad [18, 25, 26, 28–30], yet none of the above causes was statistically significant on bivariate analysis level except prematurity (P = 0.054) which was very close to significance threshold and thus exceptionally considered to go for adjustment not to miss any association. After adjustment, prematurity was independently associated with less probability of death in the NICU. It is very unique finding that contradicts the existing fact shown by studies done in Ethiopia [12], Burkina Faso [17], and Cameroon [25]. It is also in objection with another study conducted in Jordan which revealed a higher rate of neonatal mortality among the preterm neonates (123/1000 live births) compared to full term neonates (4/1000 live births) [30]. However, this finding needs to be carefully interpreted and can be partly explained by the fact that there is an improved survival of preterm neonates due to the availability of NICU—which is among the list of high impact child survival interventions implemented to reduce neonatal deaths immediately after birth–the most vulnerable period for neonates [4].

Another study in Canada argues that very preterm infants have a longer life span than all NICU patients. This delayed mortality in premature infants suggests that a greater percentage of preterm neonates are surviving from the immediate conditions of prematurity, only to contract lethal complications later[28]. A shorter duration of stay of below two days in the NICU also showed protection against neonatal mortality. This is in line with the established fact that most of the neonatal deaths occur in the first 24 hours following birth, and any intervention at this critical time has significant contribution to saving neonates [7, 12] as this period is the transition from foetal to neonatal physiology which needs appropriate support [12, 15, 29, 31].

A merit of this study is that it reviewed all neonates admitted in the NICU of the hospital ever since its establishment without sampling, hence eliminating any possible sampling error. However, as this study used secondary data, the findings are subjected to issues related to incomplete recording for which mitigation measures were taken during data collection and analysis phase. Another limitation is the relatively small number of deaths reported which have affected some of the statistical tests.

### Conclusion

The causes of neonatal death reported in this study are preventable. The neonatal mortality rate is high compared to the national and subnational figures, a prematurity and shorter length of stay in the NICU of less than two days showed protective effect against neonatal death. This calls for more work along the continuum of care by improving the quality of care, early transfer of sick neonates to the NICU along with scaling up establishment of NICUs to in other hospitals of the region.

## Supporting information

S1 File Dataset. (XLSX)

## Abbreviations

CI: Confidence Interval
AOR: Adjusted Odds Ratio
COR: Crude Odds Ratio
EMONC: Emergency Obstetric New-born Care
EDHS: Ethiopian Demographic Health Survey
MDG: Millennium Development Goal
NMR: Neonatal Mortality Rate
NICU: New-born Intensive Care Unit
RMNCH: Reproductive Maternal New-born and Child Health

## References

[1] UNICEF, W., WB, UNFPA, Levels & Trends in Child Mortality, Report 2015.

[2] UNICEF, Committing to Child Survival: A Promise Renewed 2015.

[3] UNICEF, W., WB, UNFPA, Levels & Trends in Child Mortality Report. 2014.

[4] FMOH, National Strategy for Newborn and Child Survival in Ethiopia: 2015/16-2019/20. 2015.

[5] CSA, ETHIOPIA Demographic and Health Survey 2016: Key Indicators Report 2016.

[6] Department of Maternal, N., Child and Adolescent Health (MCA/WHO), Ethiopia: Maternal and Perinatal Health Profile 2014.

[7] Central Statistical Agency, E. and M. The DHS Program ICF Rockville, USA Ethiopia Demographic and Health Survey 2011 2012.

[8] UNICEF, W., WB, UNFPA, Levels & Trends in child mortality: Report. 2017.

[9] DC B., RJ MK, and L S, *Ending preventable maternal and newborn mortality and stillbirths* the bmj 2015 BmJ 351:suppl1: p. 351:h4255 10.1136/bmj.h4255 26371222

[10] Chow S C.R., Popovic M, Lam M, Popovic M, Merrick J, et.al, A selected review of the mortality rates of neonatal intensive care units. Front. Public Health (2015). 3:225 10.3389/fpubh.2015.00225 26501049 PMC4595739

[11] UNICEF, F.a., COUNTDOWN TO A HEALTHIER ETHIOPIA: Building on Successes to Accelerate Newborn Survival. 2014.

[12] Worku B, K. A., Mekasha A, Tilahun B, Worku A *Predictors of early neonatal mortality at a neonatal intensive care unit of a specialized referral teaching hospital in Ethiopia* Ethiopian. journal of Health Dev. 2012 26(3)

[13] Worklu B., *Classification of facilities for Newborn services and the Minimum Requirements in Ethiopian setup*: *Draft guideline*. 2016. Forthcoming.

[14] UNICEF, Investing in Survival: Enhancing the Neonatal Intensive Care Unit of Yekatit 12 Hospital 2013.

[15] Gessesse, M., NICU status in Ethiopia (unpublished). 2014. Forthcoming.

[16] Costa S, R. M., Centeno MJ, Martins A, Vilan A, Brandão O, Diagnosis and cause of death in a neonatal intensive care unit – How import- ant is autopsy? J Matern Fetal Neonatal Med (2011). 2011 24(5):: p. 760–3. 10.3109/14767058.2010.520047 20945996

[17] Coulibaly A, B. A., Millogo T; Meda IB, Koueta F, Kouanda S, Predictors of mortality of low birth weight newborns during the neonatal period: A cohort study in two health districts of Burkina Faso. International Federation of Gynecology and Obstetrics, 2016. 10.1016/j.ijgo.2016.08.006 27836092

[18] Ethiopian Public Health Intitute, F.m.o.h., Columbia University New York, USA ETHIOPIAN Emergency Obstetric and Newborn Care (EmONC) Assessment 2016 2017.

[19] Pepler PT, U. D., Nel DG., Predicting mortality and length-of-stay for neonatal admissions to private hospital neonatal intensive care units: a South African retrospective study. Afr Health Sci (2012). 2012 12(2):: p. 166–73. 10.4314/ahs.v12i2.14 23056023 PMC3462546

[20] Parappil H, R. S., Salama H, Rifai HA, Parambil NK, Ansari WE, *Outcomes of 28+1 to 32+0 weeks gestation babies in the state of Qatar*: *finding facility-based cost effective options for improving the survival of preterm neonates in low income countries*. Int J Environ Res Public Health (2010) 2010 7:2526–42. 10.3390/ijerph7062526 20644688 PMC2905565

[21] Manktelow BN, S. S., Field DJ, Draper ES, Population-based estimates of in-unit survival for very preterm infants. Pediatrics (2013). 2013 131(2):e425–32. 10.1542/peds.2012-2189 23319523

[22] Ekwochi U, N. I., Nwokoye IC, Ezenwosu OU, Amadi OF, Osuorah DIC, Pattern of morbidity and mortality of newborns admitted into the sick and special care baby unit of Enugu state University Teaching Hospital, Enugu state. Niger J Clin Pract (2014). 2014 17(3):346–51. 10.4103/1119-3077.130238 24714015

[23] Musooko M, K. O., Nakimuli A, Nakubulwa S, Nankunda J, Osinde MO, et al, Incidence and risk factors for early neonatal mortality in newborns with severe perinatal morbidity in Uganda. Int J Gynaecol Obstet (2014) 2014 127:201–5. 10.1016/j.ijgo.2014.05.017 25270824

[24] Nyirasafari R, C. M., Karambizi AC, Kabayiza JC, Makuza JD, Rex Wong R et.al, Predictors of mortality in a paediatric intensive care unit in Kigali, Rwanda. 2016: p. 109–115 10.1080/20469047.2016.1250031 27922344

[25] Ndombo PK, E. Q., Tochie JN, Temgoua MN, Angong FT, Ntock FN et.al, A cohort analysis of neonatal hospital mortality rate and predictors of neonatal mortality in a sub-urban hospital of Cameroon Italian Journal of Pediatrics (2017) 2017 43:52. 10.1186/s13052-017-0369-5 PMC5460331 28583154

[26] Assefa N, L. Y., Belay B, Kedir H, and B.N. Zelalem D, et.al Neonatal mortality and causes of death in Kersa Health and Demographic Surveillance System (Kersa HDSS), Ethiopia, 2008–2013. Maternal Health, Neonatology and Perinatology 2016 2:7, 2016. 10.1186/s40748-016-0035-8 PMC4950756 27437118

[27] Mekonnen Y, T. B., Telake DS, Degefie T, Bekele A, Neonatal mortality in Ethiopia: trends and determinants. BMC Public Health 2013 13:483 10.1186/1471-2458-13-483 23683315 PMC3659057

[28] Andrew Simpson C, X.Y. Y., Hellmann J, Tomlinson C, Trends in Cause-Specific Mortality at a Canadian Outborn NICU. Pediatrics 2010 126;e153. 10.1542/peds.2010-1167 21078727

[29] Diana B, F.G., Susan G, Phyllis L., The forgotten new-born. In: Care of the new-born reference manual. Care of the new-born reference manual, 2004.

[30] Abdel Razeq Nadin M., Y.S. K., Anwar M. Batieha, The incidence, risk factors, and mortality of preterm neonates: A prospective study from Jordan (2012–2013) Turk J Obstet Gynecol 2017 14:28–36. 10.4274/tjod.62582 28913132 PMC5558315

[31] Rajaratnam JK, M. J., Flaxman AD, Wang H, Levin-Rector A, Dwyer L, *Neonatal*, *post- neonatal*, *childhood*, *and under-5 mortality for 187 countries*, *1970–2010*: *a systematic analysis of progress towards Millennium Development Goal 4*. Lancet 2010;, 2010 375(9730):1988–2008. 10.1016/S0140-6736(10)60703-9 20546887

